# Development of Nonwoven Fibrous Materials Based on Poly-3-Hydroxybutyrate with a High Content of α-Tricalcium Phosphate

**DOI:** 10.3390/polym15153167

**Published:** 2023-07-26

**Authors:** Polina M. Tyubaeva, Kristina G. Gasparyan, Alexander Yu. Fedotov, Pavel V. Lobzhanidze, Oleg V. Baranov, Alexey A. Egorov, Vladimir P. Sirotinkin, Vladimir S. Komlev, Anatoly A. Olkhov

**Affiliations:** 1 Department of Physical Chemistry of Synthetic and Natural Polymer Compositions, Emanuel Institute of Biochemical Physics, Russian Academy of Sciences, 4 Kosygina Street, 119334 Moscow, Russia; polina-tyubaeva@yandex.ru (P.M.T.);; 2 Academic Department of Innovational Materials and Technologies Chemistry, Plekhanov Russian University of Economics, 36 Stremyanny Per., 117997 Moscow, Russia; 3 A.A. Baikov Institute of Metallurgy and Materials Science, Russian Academy of Sciences, Leninsky Prospect 49, 119334 Moscow, Russiasir@imet.ac.ru (V.P.S.);

**Keywords:** poly-3-hydroxybutyrate, α-tricalcium phosphate, fibrous nonwoven material, electrospinning

## Abstract

α-tricalcium (α-TCP) phosphate is widely used as an osteoinductive biocompatible material, serving as an alternative to synthetic porous bone materials. The objective of this study is to obtain a highly filled fibrous nonwoven material composed of poly-3-hydroxybutyrate (PHB) and α-TCP and to investigate the morphology, structure, and properties of the composite obtained by the electrospinning method (ES). The addition of α-TCP had a significant effect on the supramolecular structure of the material, allowing it to control the crystallinity of the material, which was accompanied by changes in mechanical properties, FTIR spectra, and XRD curves. The obtained results open the way to the creation of new osteoconductive materials with a controlled release of the source of calcium into the living organism.

## 1. Introduction

The relevant scientific field today consists of the development of new biocompatible materials for bone and tissue engineering based on combinations of biopolymers and effective additives [1]. Of particular interest in this area is the regeneration of teeth and jaws in view of the significant disadvantages of existing methods involving auto grafts, allografts, and transplants which are expensive and painful and are accompanied by high risks of rejection [2]. The essence of the tissue engineering approach is to develop composites based on biopolymers as a biological alternative, which should not replace, but stimulate the restoration of damaged tissues [3]. Today, there are a number of successful studies on the selection and combination of biopolymers and active substances that stimulate regeneration, which include cells [4], growth factors [5], and calcium sources [6] in combination with different carbon-based carriers [7].

A large number of biocompatible polymer matrices for bone tissue regeneration based on calcium sources and collagen [8], fibroin [9], poly(lactide-co-glycolide) [10], poly(α-hydroxyl acids) [11], and polycaprolactone [12] have already been proposed. However, the question of optimizing the composition and selection of a polymer matrix with a controlled bioresorption period in the conditions of living organism is still open.

**Figure 1:**
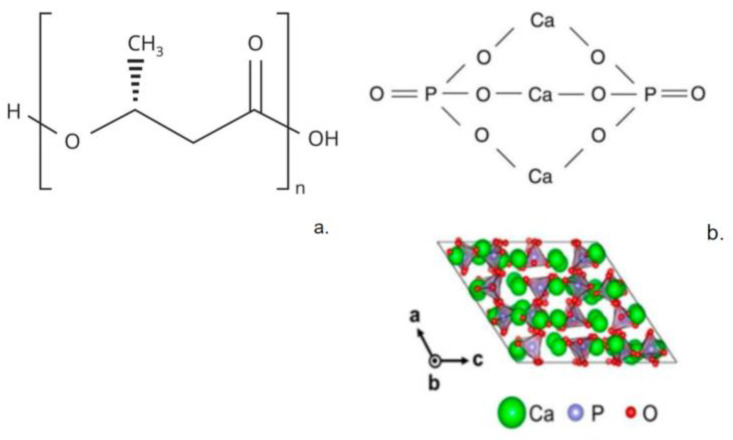
Structures of (**a**) PHB; (**b**) α-TCP [35] (Reproduced with permission from Arputharaj Joseph Nathanael et al., Crystals published by MDPI, 2021).

Particular attention among the sources of calcium should be paid to α-tricalcium phosphate (αTCP). α-TCP (Figure 1) is a form of tricalcium phosphate (Ca_3_(PO_4_)_2_). It is biocompatible, and α-TCP is a representative of calcium phosphates, which are well known as effective osteoconductive materials [13]. α-TCP-based materials in different forms, such as cements, composites, and coatings, are widely used for dental applications [14]. These materials are highly biocompatible and do not induce immunological reactions [15].

α-TCP is widely used as an osteoinductive biocompatible material, serving as an alternative to synthetic porous bone materials. Due to its porous structure, the migration of primary human osteoblasts occurs, leading to faster bone tissue regeneration [15,16]. Moreover, not only osteoinductive but also osteogenic properties of α-TCP are mentioned as for hydroxyapatite [17]. For instance, complete osseointegration based on hydroxyapatite material occurred after 4 months, while with α-TCP, it took only 6 weeks [18]. All of these advantages certainly make α-TCP very interesting for research in the field of creating new composites for regeneration.

In clinical practice, there are several types of bone grafts [19] that can be utilized for bone tissue regeneration. These include autogenous grafts (obtained from the patient’s own tissues) [20], allogeneic grafts (transplants obtained from donor bone tissue) [21], xenogenic grafts (obtained from animal tissue), and synthetic grafts (artificial materials designed to simulate bone tissue or create a supportive matrix for bone regeneration) [22]. Synthetic bone grafts have various applications in medicine and show promise. Particularly interesting are composites based on α-TCP. This material exhibits osteoinductive properties and promotes the proliferation and differentiation of osteoblasts. It can be utilized in restorative dentistry, implantology, preventive dentistry, surgery, and other fields [23]. This is because chemical analysis of enamel, dentin, and bone reveals that calcium and phosphate are the main components [24]. Another advantage of the composite nonwoven fabric is its flexibility, as it can be rolled into suitable shapes or used in layers.

As a polymer matrix for such an effective calcium source, high attention should be paid to a polymer of natural origin—poly-3-hydroxybutyrate (PHB). PHB is well known due to its high biocompatibility and controlled biodegradation in the body [25,26]. PHB (Figure 1) is a biopolymer that is naturally synthesized by certain species of microorganisms belonging to the genera *Alcaligenes*, *Azobacter*, *Bacillus*, and *Pseudomonas* [27,28]. It should be noted that PHB is a semi-crystalline, thermoplastic polymer with no cytotoxic effect [29] that easily satisfies the tasks of various methods of production and modification with a wide list of additives [30].

Electrospinning (ES) was chosen as the most promising method for obtaining PHB-α-TCP composite materials with different calcium source content [31]. Electrospun materials have proven their effectiveness in bone regeneration due to the opportunities to create a unique highly developed surface filled with tissue repair activator [32,33,34].

Therefore, the aim of the article is to explore the effect of introducing α-TCP into the polymer matrix of PHB on the morphology and structure of the electrospun materials for creating osteoinductive biocompatible material.

## 2. Materials and Methods

### 2.1. Materials

Commercial poly-3-hydroxybutyrate (PHB) was obtained from microbial synthesis (series 16F, BIOMER production, Frankfurt, Germany); crystallinity was 60%; molecular weight was 206 kDa; density was 1.248 g/cm^3^; melt flow index was 10 g/10 min (180 °C, 5 kg).

The α-tricalcium phosphate (α-TCP) was prepared by mixing ammonium phosphate salts with a concentration of 0.6 M and calcium nitrate with a concentration of 1 M under constant stirring with an overhead stirrer at 150–200 rpm. The pH value of the system in the range from 6.5 to 7.0 was maintained with an aqueous ammonia solution. The temperature of the reaction medium was maintained at 22 °C. Then, it was filtered, washed with distilled water, and dried at 90–100 °C overnight in an oven. It was fired in a furnace at a temperature of 1400 °C for 9 h in an air atmosphere at a heating rate of 5 °C/min.

At the next stage, the α-TCP powder was ground in a planetary mill in alcohol for 30–40 min at 23 rpm and room temperature, filtered, dried at room temperature for 2 h, and sifted through a sieve with a mesh size of 100 μm.

### 2.2. Methods

#### 2.2.1. Obtaining of Fibrous Materials

Fibrous composite materials based on PHB-α-TCP were obtained by electrospinning (ES). A homogeneous solution for ES was prepared by dissolving PHB in chloroform at a concentration of 7% with the addition of α-TCP at concentrations 0, 3, 10, 20, and 30%. The solutions were homogenized by ultrasonic treatment.

**Figure 2:**
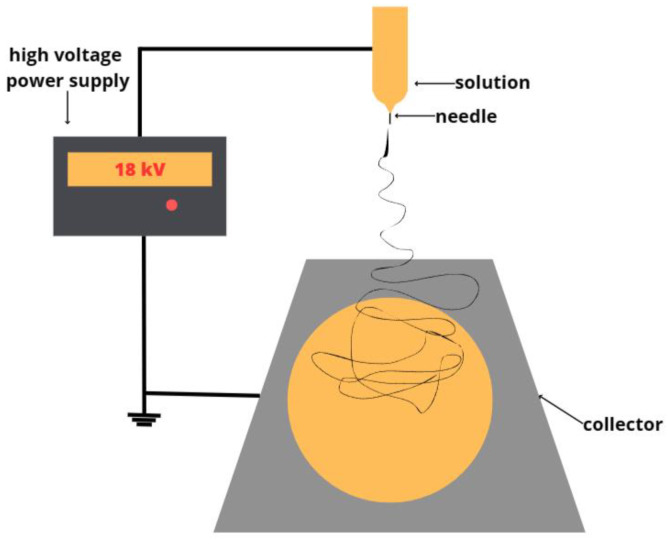
The scheme of the electroforming laboratory unit.

Fibrous materials were obtained by ES [36] using a single-capillary laboratory unit EFV-1 (IBCHP RAS, Moscow, Russia). Figure 2 shows the main elements components of the ES laboratory unit. The conditions of the ES for forming 25 mL of each solution were such that the distance between the electrodes was 250 mm, the voltage was 18 kV, and the gas pressure on the solution was 14 kg/cm^−2^. Subsequently, the materials were dried at 24 °C for 48 h to remove residual solvents and moisture.

#### 2.2.2. Optical and Scanning Electron Microscopy

Structural changes, including fiber diameter and morphological variations, were analyzed using Olympus BX43 (Olympus, Tokyo, Japan). The main morphological properties of the fibers were measured using micrography with the assistance of Olympus Stream Basic software.

Images of electrospun PHB-α-TCP composites were obtained by scanning electron microscopy (SEM) using the Tescan VEGA SBU II (Brno, Czech Republic) on the samples with gold layer.

#### 2.2.3. Surface Density

Surface density of the PHB-α-TCP composites was analyzed gravimetrically using the Balance XPR106DUHQ/A (Mettler Toledo, Columbus, OH, USA). Surface density, g/cm^3^, was calculated as follows:

(1) $$\delta =\frac{m}{l\times B\times b}$$

where *m* is the weight of the sample; *l* is the length; *B* is the width; *b* is the thickness. The average value was estimated from 10 measurements. Experimental error was below 3–5%.

#### 2.2.4. Fourier-Transform Infrared Spectroscopy (FTIR)

The IR-spectra were recorded in the 4000–400 cm^−1^ wavelength region. The KBr pellet technique was used with 1 mg of powder in 50 mg of spectroscopic-grade KBr.

#### 2.2.5. Differential Scanning Calorimetry (DSC)

Thermophysical characteristics, such as melting enthalpy, melting temperature, and degree of crystallinity, were studied using a DSC 214 Polyma (Netzsch, Selb, Germany). The DSC temperature program comprised two heating cycles (from 20 °C to 220 °C) and two cooling cycles (from 220 °C to 20 °C). The samples were tested in an argon atmosphere, with a heating and cooling rate of 10 K/min. The sample weight ranged from 6 to 7 mg.

Crystallinity degree, χ, was defined from the melting peak as follows:

(2) $$\chi =\frac{∆H}{H_{PHB}}\times 100\%\times C$$

where ∆*H* is melting enthalpy; *H_PHB_* is melting enthalpy of the ideal crystal of the PHB, 146 J/g [37]; *C* is the content of the PHB in the composition.

#### 2.2.6. X-ray Diffraction Analysis (XRD)

Phase components of samples were identified by X-ray diffraction using a TD-3700 Dandong Tongda Science and Technology (Tongda, Dandong, China) diffractometer equipped with a Mythen2 Dectris detector (CuKα radiation, tube voltage 35 kV, tube current 25 mA, step mode, step 0.04°, delay 5 s, range 8–48°.

#### 2.2.7. Mechanical Analysis

Tensile properties of composite materials were determined using a universal testing machine Instron electropuls e3000 (Instron, Norwood, MA, USA) with a load cell of 5 N capacity. Rectangular specimens of dimensions 30 mm × 5 mm were used for testing at a crosshead speed of 5 mm/min. The room conditions were controlled at 22 °C and 40% relative humidity.

## 3. Results and Discussion

### 3.1. Morphological Characterizations of Electrospun PHB-α-TCP Materials

**Figure 3:**
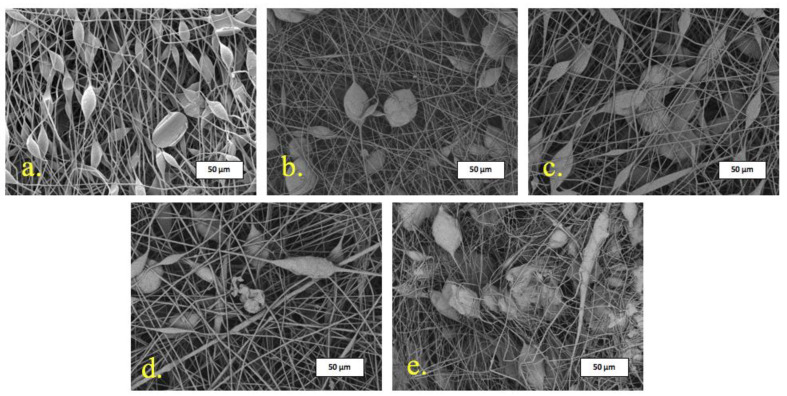
SEM images of (**a**) PHB; (**b**) PHB + 3% α-TCP; (**c**) PHB + 10% α-TCP; (**d**) PHB + 20% α-TCP; (**e**) PHB + 30% α-TCP.

Electrospinning (ES) makes it possible to obtain highly porous nonwoven materials with a high degree of surface development, which has a positive effect on tissue regeneration [38]. It is important to emphasize that ES allows to obtain a fairly uniform distribution of the additives in the material, increasing the efficiency of regeneration [39]. The features of the resulting nonwoven structure depend on a variety of parameters, including properties of polymer solution, processing parameters, and environmental conditions [40]. The introduction of additives into the polymer solution should provide sufficient values of electrical conductivity and viscosity of polymer-additive system, which also affect the voltage, flow rate, Taylor’s cone shape, and evaporation rate of the solvent. It should be noted that the introduction of even large concentrations up to 30% of α-tricalcium phosphate (α-TCP) did not interfere with the ES process, which may be due to the presence of a metal atom in the additive, which positively affects the electrical conductivity of the forming solution. SEM images of obtained materials are shown in Figure 3.

**Figure 4:**
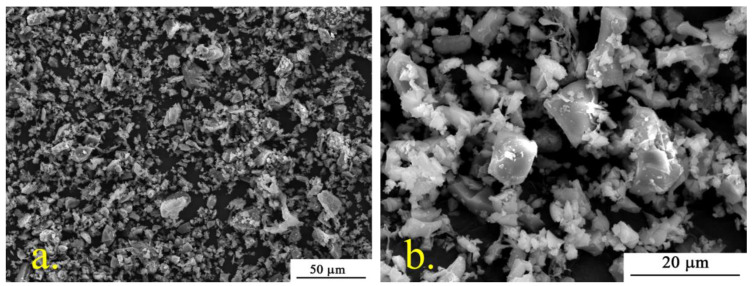
SEM images of α-TCP: (**a**)—×1000; (**b**)—×4000.

Attention should be paid to the specific pear-shaped defects represented by spindles (Figure 3a), which are a feature of the fiber formation of poly-3-hydroxybutyrate (PHB) [41]. Insufficient balance of viscosity and electrical conductivity of the solution of pure PHB leads to the formation of such thickenings. It can be seen that the introduction of α-TCP contributes to their formation. With the introduction of α-TCP, the number of defects on the surface of the fibers decreases; however, as the concentration of the additive increases, large inclusions appear, probably α-TCP. α-TCP particles consist of two fractions (1–10 µm—80% and 10–30 µm—20%) and are splintered particles (Figure 4).

There are many technological solutions to eliminate artifacts and defects of the fiber surface [42], where one of the most effective methods is the introduction of metal-containing modifying additives that increase the electrical conductivity of the molding solution [43]. In previous works, we managed to achieve high uniformity of fibers due to the control of the electrical conductivity of the polymer solution and the modifying additive [44,45]. The introduction of a calcium source, despite the content of metal atoms, did not increase the electrical conductivity sufficiently. The number of defects decreased with 3% of α-TCP. But it is important to stress that as the α-TCP concentration increased, the shape and type of defects changed. It is noticeable that new more elongated and thickened areas on the fibers have appeared (Figure 3c–e). It could be explained by the fact that a significant part of the additive is located in the fiber, occupying amorphous regions of the structure of the PHB. With a further increase in α-TCP concentration, the additive comes to the surface in the form of characteristic crystals (Figure 3d,e). In other works, α-TCP could be found on the surface of the fibrous polymeric matrix and could be clearly visible [34]. However, in the case of the obtained PHB-α-TCP matrix, it is important to note that the source of calcium is located deep in the polymer, which will allow controlling the rate of its output, ensuring control of the healing rate, and this problem exists for α-TCP and is designated as too fast and uncontrolled rate of resorption of tricalcium phosphate [14,46].

Morphology of the PHB-α-TCP materials is characterized in Table 1.

It should be noted that the introduction of 3% and 10% of α-TCP leads to a decrease in the average diameter of the fibers (Figure 3b,c). However, increasing the concentration to 20% leads to the appearance of individual thicker fibers passing through the structure of the material (Figure 3d), which cause a significant rise in the average diameter, although a large mass of fibers still remains in the range of 1.5–1.7 µm. At the same time, when a concentration of 30% is reached, such thicker fibers change their appearance (Figure 3e) and resemble defects and local thickenings in shape.

In addition, it is seen from Table 1 that the introduction of an additive reduces the surface density of the material under the same production conditions, which indicates an increase in the proportion of open pores and the growth of the surface development.

### 3.2. Chemical Characterizations of Electrospun PHB-α-TCP Materials

**Figure 5:**
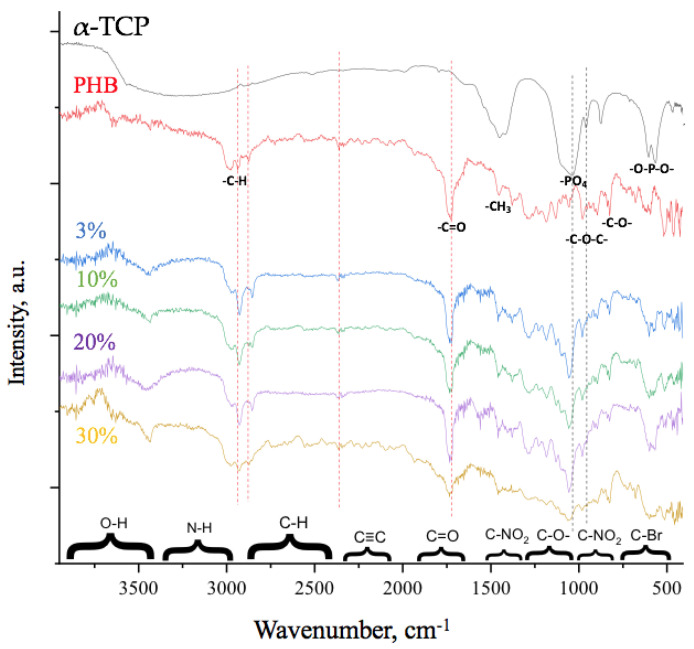
FTIR spectra of PHB-α-TCP materials.

The chemical structure of the obtained materials was determined by FTIR. FTIR spectra of PHB-α-TCP materials are shown on Figure 5.

The most pronounced chemical groups of PHB correspond to the peaks at 1721 cm^−1^ (C=O group), 1052 cm^−1^ (C-O-C group), 1278 cm^−1^ (CH_3_ group), and 3000–2700 cm^−1^ (-C-H of the main chain) [43]. As it can be seen, all of the signal characteristics for groups of PHB were detected in composite materials.

A new peak observed at 3440 cm^−1^ corresponds to adsorbed water in the materials [34]. It is known that no -OH groups could be detected in α-TCP and they are contained in very small quantities in PHB; therefore, the changes in the chemical structure of PHB chain (growth of the number of end groups of polymer chain) are assumed during the ES process. This assumption is confirmed by a decrease in the oscillation bands belonging to -PO_4_ ions at 1100 cm^−1^ and to -O-P-O- at 470 cm^−1^. Attention should be paid to the region 540–620 cm^−1^, characteristic of -O-P-O-, the intensity of which is significantly reduced as well as the decrease in the intensity of the peaks characteristic of PHB, responsible for -C-O-C- bonds at 800 cm^−1^ and at 1052 cm^−1^, which confirm the possibility of forming a chemical interaction between PHB and α-TCP [46]. In addition, intensity changes are observed in the -C-H region of the main chain, which also indicates the possibility of chemical interaction.

### 3.3. Thermophysical Properties of Electrospun PHB-α-TCP Materials

**Figure 6:**
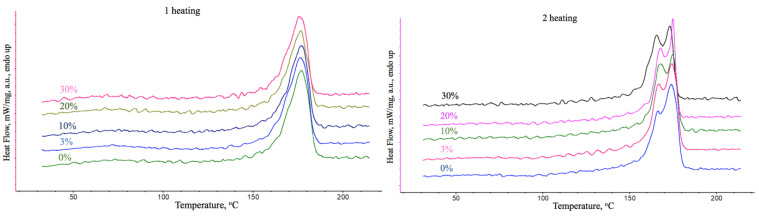
DSC curves of PHB-α-TCP materials.

The thermal properties of the obtained materials were determined by DSC. Results are shown in Table 2, and DSC curves are shown in Figure 6.

As can be seen from Table 2, the melting point of PHB changes slightly with the introduction of the additive, and the melting enthalpy decreases with the introduction of the additive. From the point of view of the organization of the supramolecular structure, the melting point allows us to estimate the size of the crystallites forming the crystalline phase of PHB. The enthalpy of melting can characterize the total fraction of the crystalline phase that passes into the melt when heated. PHB is known as a semi-crystalline polymer, which is characterized by secondary crystallization after production by pressing, watering, and 3D printing [43]. ES makes a significant contribution to the formation of the supramolecular structure of a semi-crystalline polymer [14]. In the case of PHB, ES allows for the fixing of the sections of the crystalline phase along the orientation axis of the fiber in the presence of balance of electrical conductivity and viscosity of the forming solution [47]. In addition, the presence of additives that can act as crystallization centers, especially flat morphology, has a positive effect on the formation of the supramolecular structure, which has been repeatedly observed on various modifying additives [48].

The role of α-TCP in the formation of the supramolecular structure of PHB in ES is clearly visible on the curves of DSC (Figure 6). So, in the case of the first heating, significant differences are not observed due to the role of the conditioned structure obtained by ES. However, at the second heating, the contribution of the production method is removed, and the contribution of the α-TCP could be observed [49,50,51]. The low-temperature shoulder of PHB in the range of 155–165 °C should be attributed to an irregular and poorly organized crystalline fraction of PHB, which melts at a lower temperature [52]. The fraction of this fraction will depend largely on secondary crystallization and the presence of crystallization centers and will also affect the mechanical properties of the material and the rate of biodegradation. Of course, a less organized crystal fraction undergoes bioresorption faster, since it is more accessible [53]. As can be seen from the second heating, the low-temperature shoulder is increasingly differentiated with an increase in the concentration of α-TCP, which indicates a significant effect of the additive on the crystallization process.

**Figure 7:**
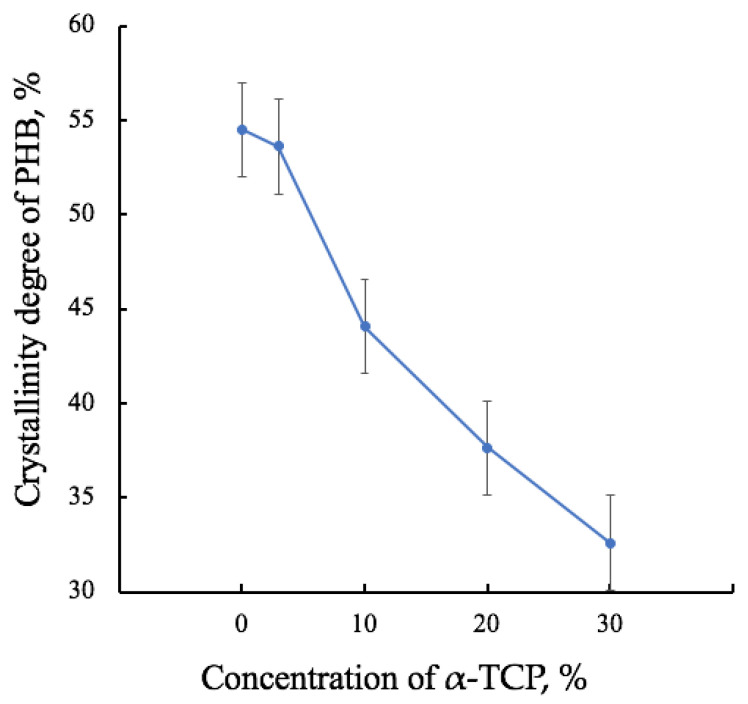
Crystallinity degree of PHB-α-TCP materials.

This effect is clearly visible when assessing the degree of crystallinity of PHB (Figure 7). Thus, there is a decrease in the degree of crystallinity by more than 20%, which is due to a significant proportion of the additive in the material.

**Figure 8:**
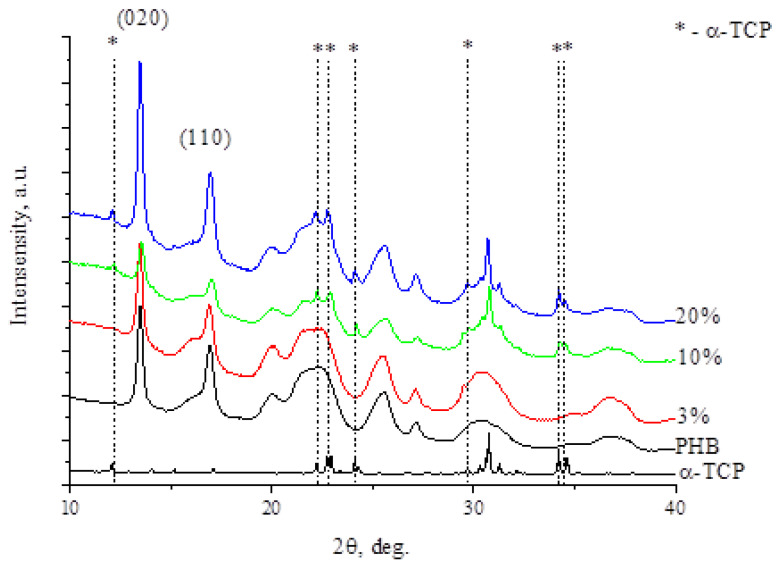
XRD curves of PHB-α-TCP materials.

These assumptions are consistent with the results of the RXD analysis, which allowed us to estimate the supramolecular structure of the PHB. XRD curves of PHB-α-TCP materials are shown in Figure 8. According to the results of the XRD analysis, it was found that the main phase is PHB (Poly(3-hydroxybutyrate) ICDD Card. № 00-049-2212.) With the introduction of α-TCP, the appearance of reflexes corresponding to α-tricalcium phosphate is observed (ICDD Card. № 01-070-0364). With increasing concentration, the intensity of reflexes of the corresponding tricalcium phosphate increases.

XRD patterns of PHB-based materials demonstrate Bragg reflections corresponding to the orthorhombic crystal lattice (P2_1_2_1_2_1_) [54], which indicates that there is no contribution of α-TCP to the formation of the chain. The characteristic peaks for PHB are 020 and 110 [55]. Thus, the ratio of peaks varies slightly and decreases from 0.65 for pure PHB to 0.5 for PHB-α-TCP with 20% of the additive, which indicates a possible small increase in the formation of PHB crystalline structure in the b-direction [56], but at the same time it corresponds to the decrease in the proportion of the crystalline phase.

### 3.4. Mechanical Properties

**Figure 9:**
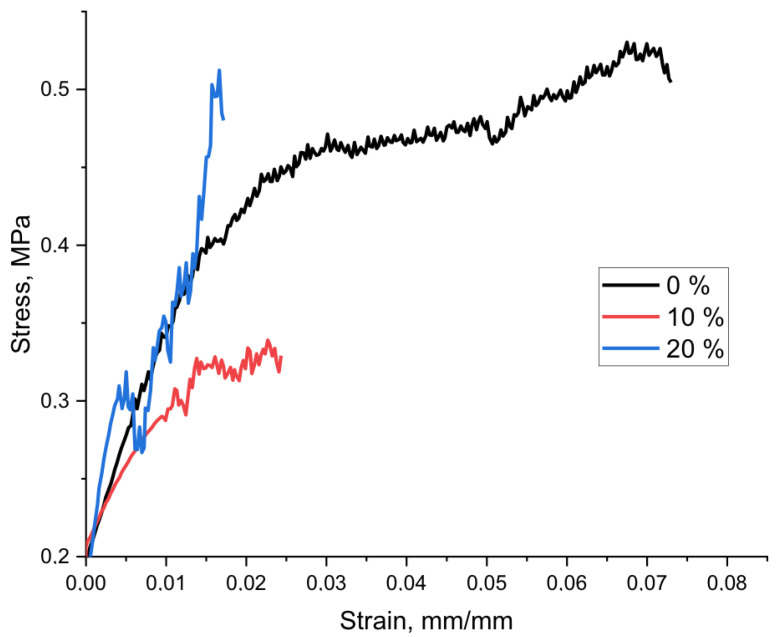
Tensile stress–strain curves of composite materials with different contents α-TCP.

**Figure 10:**
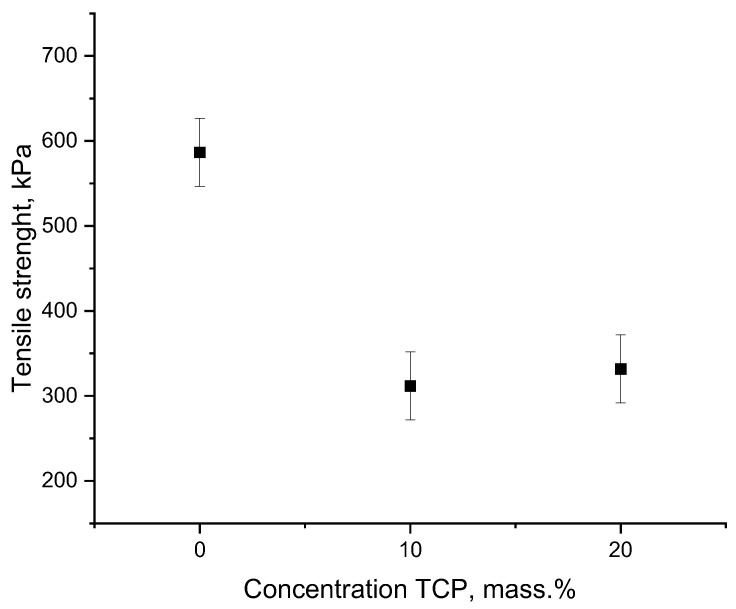
Tensile strength of composite materials with different contents α-TCP.

Tensile stress–strain curves of PHB-α-TCP are shown on Figure 9, and tensile strength of PHB-α-TCP is given on Figure 10. It must be noted that PHB-based fibrous materials are usually fragile and have low mechanical properties [57,58]. The addition of α-TCP had a significant effect on the strength of materials, reducing it by almost by a half (Figure 10). In general, this result is consistent with the changes seen in the DSC and FTIR. The crystallinity decreases; the crystal structure becomes less perfect; large inclusions of α-TCP lead to rapid destruction of materials in areas where the fibers contain the largest inclusions of the additive.

The process of deformation of a nonwoven material based on PHB represents destruction at the moment when the most durable fibers passing through the entire system do not withstand the load and are destroyed [58]. The stress–strain curves (Figure 9) also show the breaks of individual, less durable fibers, which do not lead to the destruction of the entire system. It should be noted that in the case of 20%, the shape of the curve changes significantly, where we see more abrupt discontinuities, which indicate that the number of fibers loaded with large α-TCP particles is significantly greater than in the case of 10%.

**Figure 11:**
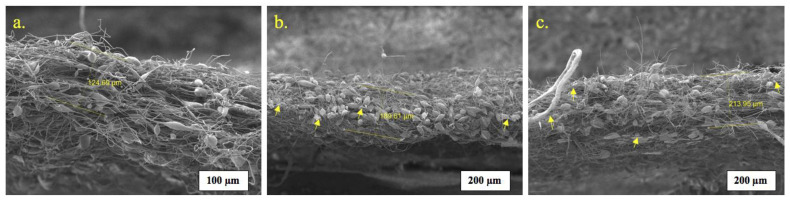
SEM images of (**a**) PHB; (**b**) PHB + 10% α-TCP; (**c**) PHB + 20% α-TCP (yellow arrows show accumulations of a calcium source).

In this case, the mechanism of rupture of the PHB-α-TCP matrices is of interest. Previously, we have established how the mechanical deformation of nonwovens electrospun materials based on PHB occurs [59]. First of all, the most stressed areas of the fibrous structure are torn [60]. In general, two aspects of the fibrous structure in such materials contribute to the strength properties of the entire material: macrodefects by which the material can tear (thickening, gluing, and snagging of fibers) and the ability of fibers to move freely relative to each other. In addition, the supramolecular structure and the degree of its filling with modifying additives also make a significant contribution. Figure 11 shows the places of rupture of fibrous materials. Yellow arrows show accumulations of a calcium source.

It should be noted that under the same molding conditions, the thickness of the fibrous layer differs, which is due to the contribution of the additive to the molding properties. It is also important that the rupture, as can be seen from microphotographs, occurs independently of these defects, since we see the passing fibers through which the rupture occurs.

Nevertheless, it is important to note that a decrease in mechanical properties is not a problem for these composites, since their use in orthodontics does not require high strength indicators but are based mainly on the ability to control the structure and control the loading of the fibers with calcium carrier particles.

## 4. Conclusions

The fibrous nonwoven material based on a poly-3-hydroxybutyrate (PHB) biopolymeric matrix with a high concentration of α-tricalcium phosphate (α-TCP) filler (0, 3, 10, 20, and 30%) was successfully produced. The composite may be used in regenerative medicine, particularly in the field of dentistry and implantology due to its high biocompatibility, bioresorbability, and osteoinductive properties. Based on the results obtained from the study, it can be concluded that the introduction of α-TCP into PHB using the electrospinning method (ES) has both positive and negative effects on the composite material. Positive effects are as follows:

- Improved supramolecular structure, with the addition of α-TCP allowing better control of its crystallinity, results in enhanced mechanical properties and structural integrity.
- The presence of α-TCP in the composite material enables a controlled release of calcium, making it a potentially suitable osteoconductive material for applications in living organisms.
- α-TCP acts as a crystallization center, positively affecting the formation of the supramolecular structure in the nonwoven fibrous material, which could lead to improved properties of nonwoven material and controlled time of resistance to biodegradation or bioresorption.

Negative effects are the formation of pear-shaped defects, large inclusions, that can potentially affect the structural integrity of the composite and appearance of thicker fibers, causing a decline in the average fiber diameter and, consequently, a decrease in mechanical properties. But it is important to stress that such effects have a very low impact on the general operational properties in the case of the creation of osteoconductive material.

It was found that the introduction of high concentrations of α-TCP can significantly affect the supramolecular structure of the PHB, providing a noticeable decrease in the degree of crystallinity of the material, while maintaining the basic parameters of the crystalline structure, which will allow the effective control of the rate of bioresorption of the implanted material and, as a consequence, the control of the rate of α-TCP output. It is important that the obtained materials have high porosity and a high degree of surface development, which is certainly an important aspect in the design of effective materials for regeneration and restoration.

Given its unique combination of properties, this composite material has the potential to revolutionize multiple industries and contribute significantly to advancements in medicine and environmental sustainability. However, further research is necessary to optimize its properties for specific applications, ensuring its safe and effective use.

## Figures and Tables

**Table 1:** Morphology of the PHB-α-TCP materials.

Sample	Concentration of α-TCP, %	Average Diameter, µm Δ ± 0.04 µm	Surface Density, g/cm^3^ Δ ± 0.04 g/cm^3^
PHB	0	2.2	0.30
PHB-α-TCP	3	1.6	0.24
PHB-α-TCP	10	1.7	0.22
PHB-α-TCP	20	2.5	0.20
PHB-α-TCP	30	1.6	0.20

**Table 2:** Thermal Properties of PHB-α-TCP materials, ∆H -melting enthalpy Δ ± 2.5%, *T_m_*—melting temperature Δ ± 2%.

Sample	Concentration of α-TCP, %	First Heating Run		Second Heating Run	
		*T_m_*, °C	Δ*H*, J/g	*T_m_*, °C	Δ*H*, J/g
PHB	0	176	79.6	174	76.8
PHB-α-TCP	3	176	80.7	174	75.0
PHB-α-TCP	10	177	71.5	175	68.1
PHB-α-TCP	20	177	68.7	175	67.7
PHB-α-TCP	30	176	68.0	173	66.5

## Data Availability

Not applicable.
